# Defects in Mitochondrial ATP Synthesis in Dystrophin-Deficient *Mdx* Skeletal Muscles May Be Caused by Complex I Insufficiency

**DOI:** 10.1371/journal.pone.0115763

**Published:** 2014-12-26

**Authors:** Emma Rybalka, Cara A. Timpani, Matthew B. Cooke, Andrew D. Williams, Alan Hayes

**Affiliations:** 1 Centre for Chronic Disease Prevention and Management, College of Health and Biomedicine, Victoria University, Melbourne, Victoria, Australia; 2 Institute of Sport, Exercise & Active Living (ISEAL), Victoria University, Melbourne, Victoria, Australia; 3 Australian Institute of Musculoskeletal Science, Western Health, Victoria, Australia; 4 School of Human Life Sciences, University of Tasmania, Launceston, Australia; University of Minnesota, United States of America

## Abstract

Duchenne Muscular Dystrophy is a chronic, progressive and ultimately fatal skeletal muscle wasting disease characterised by sarcolemmal fragility and intracellular Ca^2+^ dysregulation secondary to the absence of dystrophin. Mounting literature also suggests that the dysfunction of key energy systems within the muscle may contribute to pathological muscle wasting by reducing ATP availability to Ca^2+^ regulation and fibre regeneration. No study to date has biochemically quantified and contrasted mitochondrial ATP production capacity by dystrophic mitochondria isolated from their pathophysiological environment such to determine whether mitochondria are indeed capable of meeting this heightened cellular ATP demand, or examined the effects of an increasing extramitochondrial Ca^2+^ environment. Using isolated mitochondria from the diaphragm and tibialis anterior of 12 week-old dystrophin-deficient *mdx* and healthy control mice (C57BL10/ScSn) we have demonstrated severely depressed Complex I-mediated mitochondrial ATP production rate in *mdx* mitochondria that occurs irrespective of the macronutrient-derivative substrate combination fed into the Kreb’s cycle, and, which is partially, but significantly, ameliorated by inhibition of Complex I with rotenone and stimulation of Complex II-mediated ATP-production with succinate. There was no difference in the MAPR response of *mdx* mitochondria to increasing extramitochondrial Ca^2+^ load in comparison to controls, and 400 nM extramitochondrial Ca^2+^ was generally shown to be inhibitory to MAPR in both groups. Our data suggests that DMD pathology is exacerbated by a Complex I deficiency, which may contribute in part to the severe reductions in ATP production previously observed in dystrophic skeletal muscle.

## Introduction

Duchenne Muscular Dystrophy (DMD) is a fatal neuromuscular disease characterised by progressive fibre necrosis secondary to the absence of the protein dystrophin from the sarcolemma [1]. This leads to severe muscle wasting and weakness, and eventually death in all patients afflicted with the disease, usually by the third decade of life [2]. A prominent yet commonly ignored feature of DMD is compromised bioenergetical status. A 50% deficit in resting ATP levels in dystrophic skeletal muscle has been reported [3], [4] which is likely reflective of both an increased demand for calcium (Ca^2+^) buffering, satellite cell cycling and muscle regeneration, alongside an inability of cellular energy systems to match this heightened demand with sufficient ATP production. Indeed, functional aberrations in key intracellular energy systems, including the mitochondria, have been consistently reported in the literature [4]–[8]. It is likely that these aberrations are strongly associated with the drastically increased intracellular [Ca^2+^] that is observed in dystrophin-deficient myofibres [9], and contribute significantly to the muscle wasting phenotype of DMD.

Mitochondria are important regulators of [Ca^2+^]_i_ in skeletal muscle and work synchronously with the sarcoplasmic reticulum (SR) to maintain a resting [Ca^2+^]_i_ of approximately 50 nM, and handle 100-fold functional oscillations of up to 5 µM during excitation-contraction coupling. Increasing [Ca^2+^]_mit_ during activity is thought to provide functional benefits to the muscle whereby oxidative ATP production can be matched to demand at the cross-bridge level [10], [11]. Ca^2+^ is thus considered a positive stimulator of oxidative phosphorylation (OXPHOS) [10]–[13] and in dystrophic muscle where resting [Ca^2+^]_i_ is demonstrably two-fold higher [14]–[16] the stimulus for mitochondrial ATP production should, theoretically, be elevated.

A reduced capacity for OXPHOS by dystrophic mitochondria has, however, been consistently reported in the literature [5]–[8], [17]–[20], Assessment of O_2_ consumption indicates a 30–35% decrease in mitochondrial respiration rates of both type I and type II fibres [8], [21] and *mdx* myoblasts [7] yet it is unclear as to whether reduced OXPHOS capacity is attributable to any specific metabolic pathway or intrinsic to the mitochondria. Chi *et al*. [22] has reported impaired glycolysis and hence carbohydrate metabolism, but speculate that increased fatty acid metabolism adequately compensates this deficit. Conversely, Kuznetsov *et al*. [6] report a 50% reduction in electron transport chain (ETC) enzyme activity but no change in glycolysis or TCA cycling rates, whilst Even *et al*. [5] report normal glycolytic but abnormal TCA activity.

A reason for these fundamentally different results might be that mitochondria are particularly adaptive to the environment in which they exist, and that the metabolic studies performed thus far have failed to ascertain the functional and/or pathophysiological status of the fibres/cells/myotubes that assays have been performed upon. In particular, mitochondrial function is highly dependent upon the calcium status of the cell such that highly necrotic fibres/cells are likely to have a large proportion of mitochondria that are overloaded with calcium and morphologically and/or functionally altered. As such, isolated mitochondria preparations are useful assay platforms for the investigation of disease states as the complex pathology-induced environment within the cell is effectively removed allowing intrinsic defects and/or persistent adaptations within the mitochondria to be determined. Removing mitochondria from the cellular environment allows dysfunction specific to the mitochondria to be determined, and for artificial environments around the mitochondria to be created to determine the impact of isolated variables on OXPHOS.

The present study is the first to directly quantify ATP production by dystrophic *mdx* mouse skeletal muscle mitochondria using a biochemical luciferin/luciferase reporter system. In particular, mitochondria isolated from diaphragm - which unlike hind limb muscle, parallels the progressive severity evident in human DMD [23] – was assessed. We aimed to characterise the effect of TCA substrate manipulation and increasing environmental [Ca^2+^] on mitochondrial ATP production rate (MAPR) to determine if *mdx* mitochondria respond normally to an artificially induced-dystrophinopathic (i.e. high [Ca^2+^]_i_) environment.

## Materials and Methods

### Ethics Statement

All experiments were approved by the Animal Ethics Experimentation Committee, Victoria University (AEETH 07/02), and conformed to the Australian Code of Practice for the Care and Use of Animals for Scientific Purposes (7^th^ edition, National Health and Medical Research Council (NHMRC), 2004). Animals were housed in pairs and provided enrichment, and all procedures were conducted to maximise animal welfare by following the Guidelines to Promote the Wellbeing of Animals used for Scientific Purposes (NHMRC, 2008).

### Animals

Age-matched normal C57BL/10ScSn (13.17±0.21 weeks) and dystrophic *mdx* (12±0.5 weeks) mice were sourced from the Animal Resources Centre (Western Australia, Australia), housed at the Victoria University Animal Facility (Werribee Campus, Victoria, Australia) on a 12∶12 hour light-dark cycle and were permitted *ad libitum* access to food and water. A total of 36 animals were used in this study: For MAPR, CS function, and mitochondrial protein experiments n = 10 for both control and *mdx* groups; for the mitochondrial swelling experiment n = 8 for both control and *mdx* groups.

The diaphragm was selected to study the full range of metabolic substrates in this study due to its human-DMD comparative phenotype [23], whereas a smaller subset of substrates (PPKM and S+R) was used to assess TA. On the day of experimentation, animals were anaesthetised (sodium pentobarbitone; 60 mg/kg; IP) and both muscle were excised for mitochondrial isolation.

### Mitochondrial Isolation

For the MAPR assay, mitochondria were isolated according to adapted methods of Wibom *et al.* [24] and is described briefly. Muscles were dissected free of connective tissue, finely minced and ground-glass homogenised in Solution A (KCl 100 mM; TRIS 50 mM; MgCl_2_.6H_2_O 5 mM; ATP 1.8 mM; EDTA 1 mM; pH 7.2) (M1). M1 homogenate was centrifuged (650 G, 4°C, 3 mins) and the supernatant retained and re-centrifuged (15000 G, 4°C, 3 mins). The mitochondrial pellet was retained and re-suspended in Solution A with graded fine-bore glass pasteur pipettes and re-centrifuged (15000 G, 4°C, 3 mins). This step was repeated. The final pellet was re-suspended in 200 µL mitochondrial storage solution (200 µl, Solution B containing: sucrose 180 mM; KH_2_PO_4_ 35 mM; Mg Acetate 5 mM; EDTA 1 mM; pH 7.5 with KOH) to yield the final mitochondrial suspension (M2). M2 suspension was diluted 1∶5 with Solution B and a volume portion was snap frozen for later determination of mitochondrial protein content, a volume retained on ice for determination of citrate synthase activity and the remaining volume further diluted 1∶5 with ATP monitoring reagent containing firefly luciferase (AMR; containing: sucrose 180 mM; KH_2_PO_4_, 35 mM; Mg acetate, 5 mM; EDTA, 1 mM; Na_4_P_2_O_7 _mM; and 1% BSA, pH 7.5 with KOH; reconstituted FL-AAM ATP assay mix (Sigma Chemical Company, MO, USA) containing luciferase and luciferin) to yield the working mitochondrial suspension (MS). MS was used immediately to measure ATP production rate.

### Measurement of Mitochondrial ATP Production Rate (MAPR)

Substrate cocktails of pyruvate and malate (1 mM each; P+M); palmitoyl-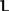-carnitine (0.005 mM) and malate (10 mM) (PC+M); α-ketoglutarate (10 mM; α-KG); pyruvate (1 mM), palmitoyl--carnitine (0.005 mM), α-ketoglutarate (10 mM) and malate (10 mM) (PPKM); and succinate (20 mM and rotenone (0.1 mM) were prepared in AMR solution with a final [ADP] of 0.04 mM to stimulate the various entry points for substrate channelling into the TCA to fuel OXPHOS.

To test the effects of increasing extra-mitochondrial [Ca^2+^] on MAPR, a series of stock CaCl_2_ solutions were prepared to give final concentrations in the substrate cocktails of 50, 100, 200 and 400 nM, respectively. To determine the effects of [Ca^2+^] on MAPR for both diaphragm and TA, only PPKM and S+R substrates were utilised as these substrates give a good indication of how each of NADH- and FADH_2_-stimulated OXPHOS is affected by Ca^2+^, respectively.

MAPR was determined using firefly luciferase (Sigma, Australia) according to the methods of Wibom *et al*. [25] as previously used in our laboratory [26], [27]. Each well was calibrated with an internal ATP standard at the commencement of the assay. Basal ATP production was measured and ATP synthesis induced via the addition of ADP. [26], [27]. For all experiments, a background assay was performed containing mitochondria with no substrate to determine non-mitochondrial ATP production via the adenylate kinase and other non-specific reactions (see S1 Table). Linear, time-dependent ATP production was confirmed for all assays. Data is the average MAPR of triplicate readings and corrected for intact mitochondrial yield (%) as determined by sequential citrate synthase activity analysis. Control (n = 10) and *mdx* (n = 10) group sizes were utilised for these experiments.

### Measurement of Citrate Synthase

CS synthase activity was measured spectrophotometrically (412 nm, 25°C), according to the methods of Srere [28]. CS activity was measured in the original mitochondrial extract on the day of experimentation post-MAPR assay (CS_before_), in the original mitochondrial extract containing 1% Triton-X after periodical snap-freeze and -thaw cycles to encourage fracture and disintegration of the mitochondrial membranes and CS liberation (CS_after_), and in a separate snap-frozen sample of the same muscle on which MAPR was performed after mechanical homogenisation and complete disintegration of sub-cellular structures (CS_total_).

CS_after_ and CS_total_ samples were stored and homogenised in homogenising buffer (KCl 175 mM and ethylenediaminetetra-acetic acid (EDTA) 2 mM; pH 7.4) and activity was calculated utilising the extinction coefficient for CS of 13.6 [28]. The proportion of CS activity in CS_before_, CS_after_ and CS_total_ samples was used to calculate intact mitochondrial yield for correction of MAPR.

### Measurement of Mitochondrial Proteins

Mitochondrial protein content was analysed on snap frozen samples derived from MS using a Bradford Protein Assay kit in triplicate (Bio-Rad Protein Assay, Bio-Rad Laboratories, Hercules, CA, USA). Mitochondrial protein was used to correct for CS activity.

### Measurement of Mitochondrial Swelling

Mitochondrial swelling was measured spectrophotometrically in a microplate assay adapted from [29]. 5 µL of diaphragmatic MS (∼10 µg mitochondrial protein) was added to each well and the assay was initiated via the addition of 90 uL of Solution B containing glutamate, malate and succinate (G+M+S; all 10 mM; [30]). A baseline reading was recorded for 4 mins (excitation wavelength = 490 nm; emission wavelength = 525 nm) before the addition of 5 µL of vehicle control (G+M+S in Solution B) or the appropriate [Ca^2+^]. An additional 30 µM [Ca^2+^] was utilised as a positive control as a known activator of the mitochondrial permeability transition pore inducing maximal mitochondrial swelling subsequent to bursting. Mitochondrial swelling was followed for 16 minutes, with a decrease in absorbance indicating increased size of the mitochondria (n = 8 for both groups for these experiments).

### Statistics

Results are expressed as mean ± standard error of the mean. [Ca^2+^]-treated MAPR data is expressed relative to MAPR at 0 nM [Ca^2+^]. A three-way analysis of variance (ANOVA) was utilised to detect between group, substrate and muscle differences in MAPR at 0 nM [Ca^2+^], and between group, [Ca^2+^] and muscle differences in MAPR across the [Ca^2+^] range. A two-way ANOVA was utilised to detect between group and muscle differences for CS function, mitochondrial protein concentration and mitochondrial yield in both TA and diaphragm. A two-way ANOVA was utilised to detect between group and [Ca^2+^] difference in mitochondrial morphology. Subsequent post hoc analysis using Tukey’s test was utilised to identify interactions between the variables. An α value of 0.05 was considered significant.

## Results

### MAPR of *mdx* mitochondria from diaphragm and TA

**Figure 1:**
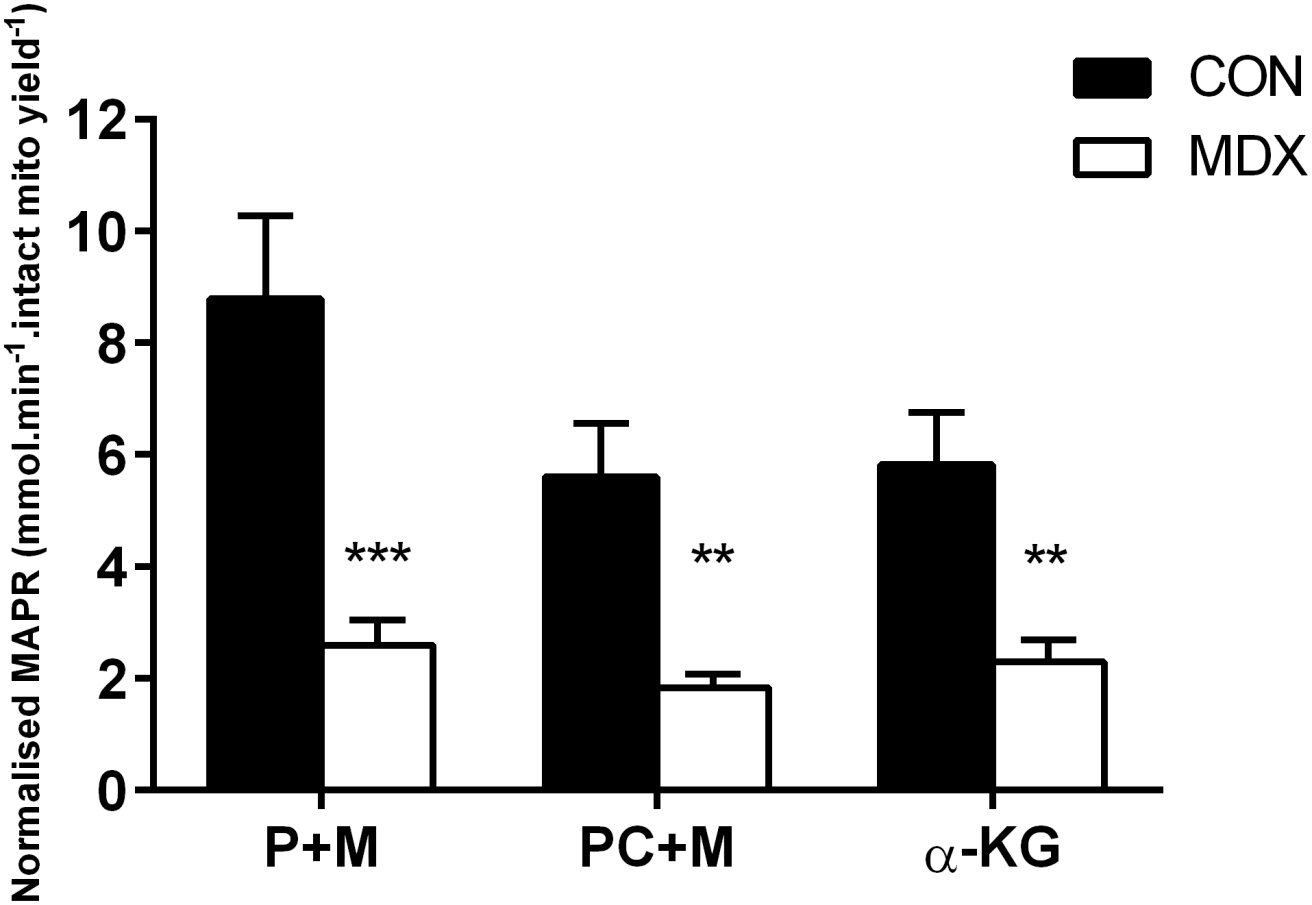
MAPR of normal (control C57BL/10) and dystrophic *mdx* diaphragm at 0 nM extra-mitochondrial [Ca^2+^] following pyruvate and malate (P+M), palmitoyl carnitine and malate (PC+M) and α-ketoglutarate (α-KG) stimulation. ***p<0.005 from controls; **p<0.01 from controls; n = 10 control and n = 10 *mdx*.

**Figure 2:**
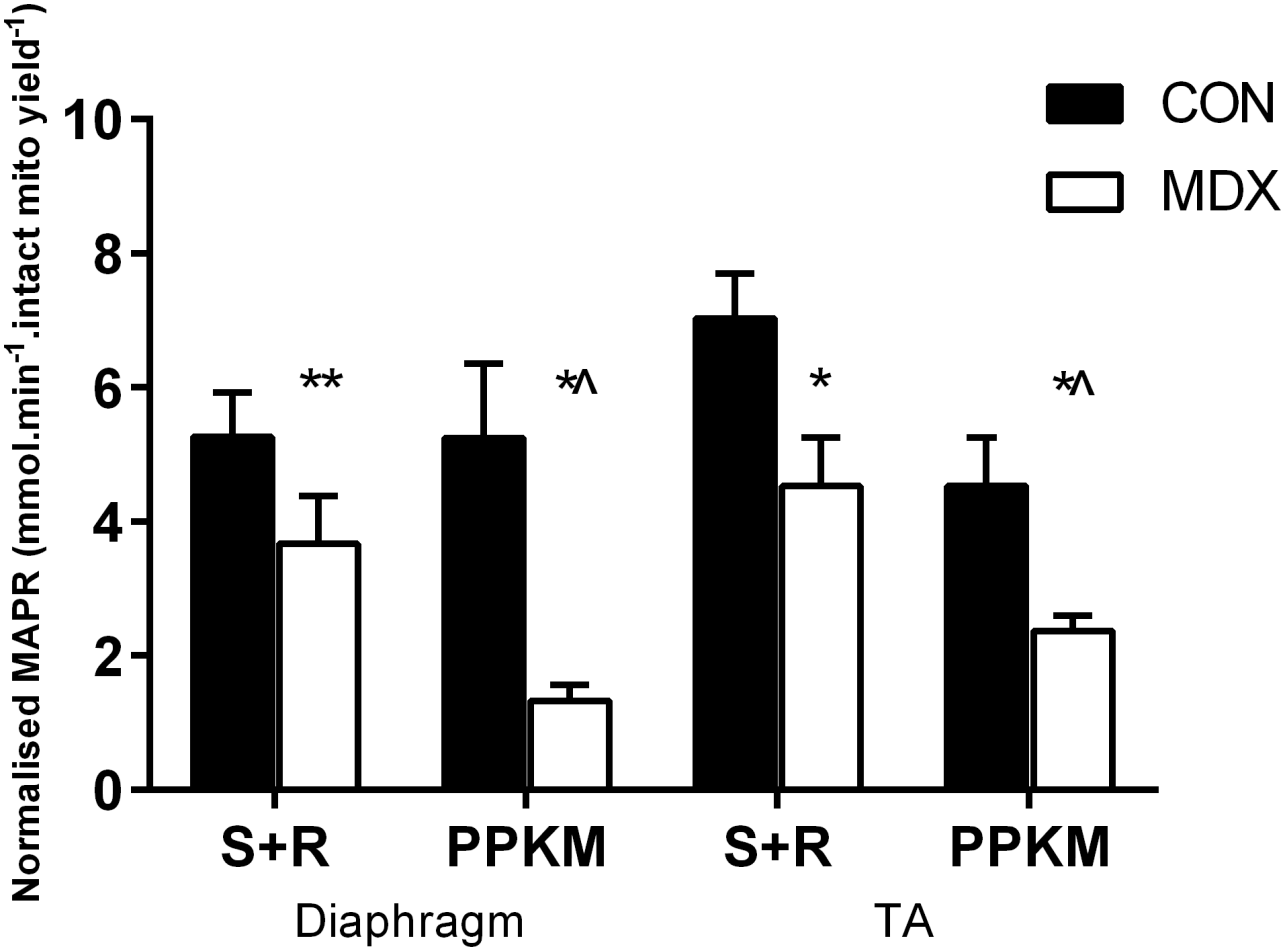
MAPR of normal (control C57BL/10) and dystrophic *mdx* diaphragm and TA at 0 nM extra-mitochondrial [Ca^2+^] following pyruvate + palmitoyl carnitine + α-ketoglutarate + malate (PPKM) and succinate + rotenone (S+R) stimulation. **p<0.01 from control group; *p<0.05 from control group;^ ∧^p<0.05 from other substrates in *mdx* group; n = 10 control & n = 10 *mdx*.

This study notably demonstrates that NADH-dependent MAPR is severely depressed in *mdx* mitochondria isolated from diaphragm (Figs. 1 and 2; p<0.001) and TA (Fig. 2; p<0.05) compared to controls and that this depression occurs irrespective of the oxidizing substrate used to stimulate OXPHOS. Stimulation of various substrate entry points within the Kreb’s Cycle that mimic the entry points for glycolytic (P+M), β-fat oxidation (PC+M) and anaplerotic glutamate oxidation (α-KG) derivatives all produced chronically depressed MAPR in diaphragmatic *mdx* mitochondria that was 70% (p<0.005), 67% (p<0.01) and 60% (p<0.01) less than controls, respectively. A similar 75% depression in diaphragm MAPR was observed when Kreb’s cycle substrates were combined (PPKM) to stimulate OXPHOS (Fig. 2; p<0.01). This effects was also observed for TA MAPR, which was 62% less than controls following PPKM stimulation (p<0.05; Fig. 2). Succinate-induced FADH_2_-dependent OXPHOS, however, significantly ameliorated MAPR of mdx diaphragmatic and TA mitochondria when compared to the NADH-generating PPKM substrate combination (p<0.05), albeit was still 30% less than control levels (p<0.05). Interestingly, this effect of the S+R combination on MAPR was not observed in the control group (p>0.05).

### Effects of a “resting” extramitochondrial [Ca^2+^] spectra on MAPR

**Figure 3:**
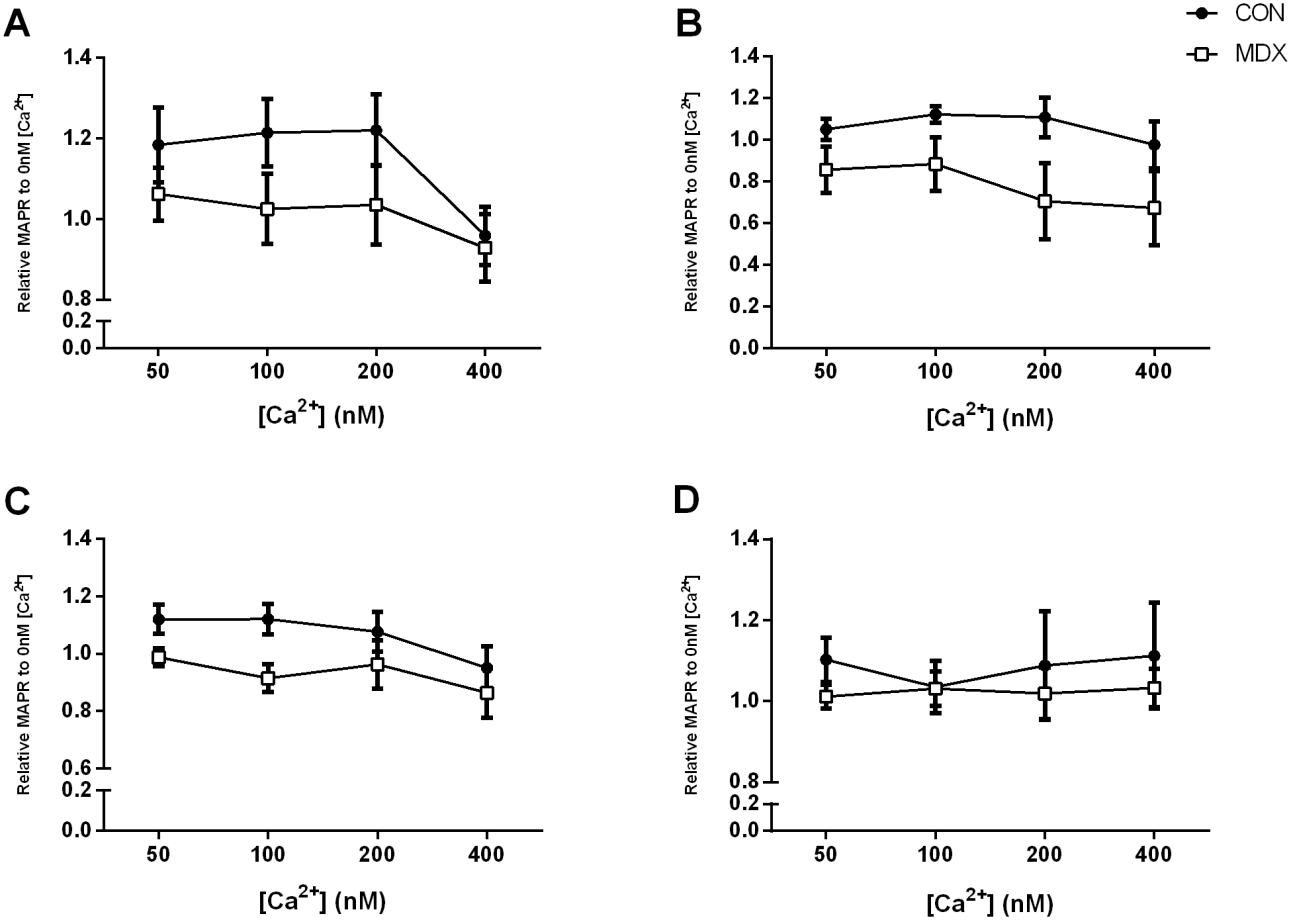
Relative MAPR of normal (control C57BL/10) and dystrophic *mdx* diaphragm following (A) pyruvate, palmitoyl carnitine, α-ketoglutarate and malate (PPKM) stimulation; (B) succinate and rotenone (S+R) stimulation; and TA following (C) PPKM stimulation; and (D) S+R stimulation across a 50–400 nM [Ca^2+^] range. Data is expressed relative to MAPR at 0 [Ca^2+^] as mean ± SEM. *p<0.05 400 nM different from other [Ca^2+^]; n = 10 control & n = 10 *mdx*.

Fig. 3 shows the effect of a 50–400 nM extra-mitochondrial [Ca^2+^]_em_ (normalised to 0 nM [Ca^2+^]), on PPKM- and S+R-stimulated MAPR in control and *mdx* mitochondria extracted from the diaphragm and TA. Overall, there was no significant effect of [Ca^2+^] on MAPR of *mdx* mitochondria when compared to controls (p>0.05 group effect). There was a trend towards reduced stimulation of PPKM-fuelled MAPR in *mdx* compared to control mitochondria from both diaphragm (*p* = 0.141; Fig. 3A) and TA (*p* = 0.069; Fig. 3C) upon addition of Ca^2+^_em_ however this observation was not concentration-specific. Irrespective of group, 400 nM [Ca^2+^]_em_ inhibited PPKM-stimulated MAPR of diaphragmatic mitochondria compared to all other [Ca^2+^]_em_ (all p<0.05); Fig. 3A). A similar effect was observed for TA mitochondria, with 400 nM Ca^2+^ inhibiting PPKM-stimulated MAPR compared to 50 nM and 100 nM (both p<0.05) but not 200 nM [Ca^2+^]_em_ (*p*>0.05; Fig. 3C).

For S+R-stimulated MAPR in diaphragm, there again was a trend towards relative Ca^2+^-induced MAPR depression (from 0 nM MAPR) in *mdx* compared to control mitochondria (*p* = 0.074; Fig. 3B). This trend was notably absent in TA mitochondria (*p* = 0.435 *mdx* versus control; Fig. 3D). There was no effect of extra-mitochondrial [Ca^2+^] on S+R-stimulated MAPR at any of the assayed concentrations for TA mitochondria, irrespective of group (Fig. 3D). In diaphragm, a 400 nM extramitochondrial [Ca^2+^] was inhibitory to S+R-induced MAPR compared to 50 nM and 100 nM [Ca^2+^]_em_ (both *p*<0.05) but not to 200 nM (*p*>0.05; Fig. 3D).

### Citrate Synthase activity

All data for CS function is described in Table 1. CS_before_ activity (a measure of free CS availability from damaged mitochondria in the MS) was 40% lower in *mdx* mitochondria from TA and diaphragm compared to controls (*p*<0.01). CS_after_ activity (a measure of the total number of mitochondria that were brought through the mitochondrial extraction process whether intact or damaged), was reduced by ∼30% in *mdx* TA and diaphragm (*p*<0.01) from controls. No difference was observed between control and *mdx* groups in the CS_before_:CS_after_ ratio, a marker of the robustness of the extracted intact mitochondria that were available to OXPHOS and therefore MAPR measurement. In a separate homogenised sample of the same muscle from which mitochondria were isolated, CS_total_ activity was unchanged between control and *mdx* groups in both TA and diaphragm. A significant decrease in the CS_after_:CS_total_ ratio (*p*<0.01), which represents the proportion of possible mitochondria that are able to withstand the extraction procedure, was observed in *mdx* muscles, in both diaphragm and TA. Mitochondrial yield (% of successfully extracted mitochondria in the MS compared to the total number of mitochondria available for extraction in the same mass of muscle) was markedly (35%) higher in control compared to *mdx* MS (*p*<0.01).

**Table 1:** Citrate synthase (CS) function and mitochondrial yield (% of total available) and protein content (% of total) of control (c57BL/10) and dystrophic *mdx diaphragm* and TA.

	CON		MDX		p values	
	TA	DIA	TA	DIA	Strain	Muscle
CS_before_ (mmol.mg^−1^ mitochondrial protein.min^−1^)	1.3±0.2	1.6±0.3	0.8±0.1	0.9±0.092	0.004**	0.338
CS_after_ (mmol.mg^−1^ mitochondrial protein.min^−1^)	11.8±0.9	11.7±0.8	8.7±0.6	8.3±1.5	0.002**	0.282
CS_total_ (mmol.mg^−1^ mitochondrial protein.min^−1^)	55.8±3.2	67.2±4.4	53.9±4.6	64.4±5.6	0.395	0.071
CS_after_:CS_total_ Ratio	0.20±0.01	0.15±0.02	0.17±0.02	0.13±0.02	0.009**	0.008**
CS_before_:CS_after_ Ratio	0.10±0.02	0.12±0.02	0.09±0.01	0.13±0.02	0.900	0.063
Mitochondrial Yield (%)	18.0±1.5	13.9±1.1	15.5±2.1	10.1±1.3	0.012*	0.009**
Mitochondrial Protein Content (% of total)	21.6±2.2	22.1±0.9	17.5±0.8	31.3±2.6	0.499	0.269

*p<0.05 from control group, **p<0.01 from control group; n = 10 control & n = 10 *mdx* for TA and diaphragm.

Comparison of CS function between muscles was also made due to the marked differences in disease progression between muscles of the lower limb and diaphragm in *mdx* mice. Mitochondrial yield was ∼20% higher in TA than in diaphragm (*p*<0.05) as was the CS_after_:CS_total_ ratio (*p*<0.01). There was a strong trend toward increased CS_total_ activity of the diaphragm compared to TA in both control and *mdx* muscle (20%; *p* = 0.07), however there were no significant differences in any of the other CS measures between muscles (CS_before_, CS_after_ and CS_before_:CS_after_ all *p*>0.05).

### Mitochondrial Morphology

**Figure 4:**
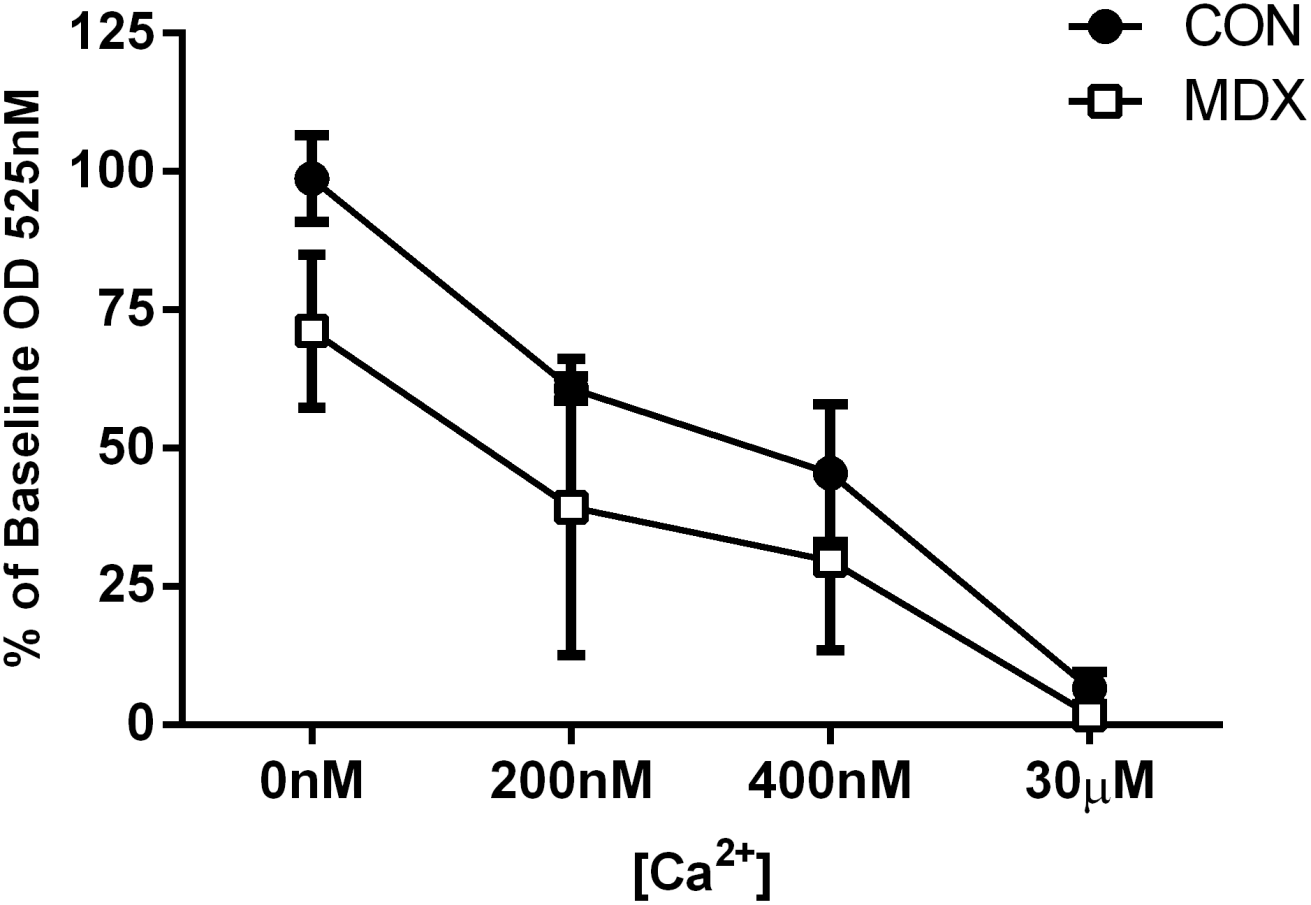
[Ca^2+^]-dependent swelling of normal (control C57BL/10) and dystrophic *mdx* diaphragm mitochondria respiring on glutamate, malate and succinate (GMS). Data is expressed as the percentage change from baseline OD at 525 nm. *p<0.05 different from control at all [Ca^2+^]; **p<0.001 all different from 0 nM [Ca^2+^] n = 8 control & n = 8 *mdx*.

*Mdx* mitochondria were significantly larger (more swollen) as reflect by a reduced % change from baseline optical density at a 525 nm wavelength in both a Ca^2+^ free environment and when exposed to increasing [Ca^2+^]_em_ (p<0.05; Fig. 4). Irrespective of strain, increasing [Ca^2+^] induced concomitant increases in mitochondrial size (p<0.05). Notably, there was large variation in the response of *mdx* mitochondria to increasing Ca^2+^_em_ load that was not evident in control mitochondria.

## Discussion

This study confirms compromised ATP production by *mdx* mitochondria (MAPR), and in particular, in mitochondria respiring on optimal and continuous delivery of oxidising substrates. This is an important finding because mitochondria have been extracted from muscle at a relatively stable period of disease progression (12 weeks) and thus, the pathophysiology and concomitant inflammatory nature of the disease is less influential. This suggests an inherent deficit of the mitochondria, which is likely reflective of strong and persistent inhibition or structural abnormality of key mitochondrial machinery leading to deficient OXPHOS. While potentially induced by the extreme pathological environment in which the mitochondria reside *in vivo*, a mounting collection of evidence suggests a natural history of inherited metabolic impairment alongside dystrophin deficiency. Onopiuk *et al.* [7] has demonstrated that metabolic dysfunction is present in dystrophic myoblasts prior to the time of dystrophin expression [7]. This suggests that while dystrophin-deficiency induced pathophysiology may exacerbate mitochondrial dysfunction, metabolic impairment exists beforehand. That female carriers of DMD who do not express the disease exhibit abnormal muscle energy metabolism, especially when ATP demand is increased during exercise, lends further credence to this notion [31], [32].

Whilst several groups have demonstrated depressed oxygen consumption rate and isolated mitochondrial enzyme function in dystrophic skeletal muscle [5]–[8], [18], [21], [22], [33], we have highlighted the differential contributions of Complex I and II H^+^ flux into the ETC and resultant ATP production in dystrophic mouse mitochondria. Importantly, these impairments were shown in the ‘healthy’ mitochondria that survived the isolation process. MAPR was shown in this study to be severely depressed by up to 75% of control levels in both diaphragm (Figs. 1 and 2) and TA (Fig. 2) mitochondria from the *mdx* mouse. MAPR depression was more evident when stimulated by substrates that enter the TCA cycle (P+M, PC+M, α-KG and PPKM) and rely on NADH-mediated shuttling of H^+^ into the ETC through Complex I. In contrast, the complete inhibition of Complex I with rotenone and stimulation of Complex II-mediated MAPR with succinate (S+R) partially ameliorated *mdx* MAPR, albeit depression was still evident (Fig. 2). This suggests a problem with NADH flux into the ETC of *mdx* mitochondria, whereby NADH is either being sequestered away from, or is unable to be efficiently oxidised by, Complex I to establish proton motive force. In this instance, the accumulation of NADH at Complex I would be inhibitory to all dehydrogenases of the Krebs cycle except for succinate dehydrogenase (including pyruvate, isocitrate, α-KG dehydrogenase)(as reviewed in [34]), which would explain why succinate stimulation was able to partially restore MAPR of mdx mitochondria closer to control levels, whereas other Kreb’s substrates had no effect. Recent literature has demonstrated reduced Complex I activity in permeabilised skeletal muscle from *mdx* mice [33] and in *mdx* brain, in which dystrophin is normally expressed but is also notably absent in DMD [35]. The expression of various Complex I subunits is also evident in *mdx* skeletal muscle at the protein level (∼1.5 fold decrease), in human DMD skeletal muscle at the transcript level (3–6 fold decrease) [36] and in *mdx* cardiac muscle. Thus our data together with others [20], [33] suggests that a persistent impairment of Complex I function underpins dystrophic pathology, which strongly limits – but does not obliterate – the ATP-producing capacity of mitochondria. While Godin et al. [33] suggests that reduced mitochondrial biomass (i.e. Complex expression/activity) underscores loss of ETC function rather than specific respiratory impairment, our data would suggest the opposite as the stimulation of respiration with succinate following Complex I inhibition with rotenone partially restored MAPR in mdx mitochondria with equivalent biomass.

It has been widely documented that [Ca^2+^]_mit_ is a positive stimulator of OXPHOS (reviewed in [37], [38]), but despite dramatic elevations of [Ca^2+^]_mit_ within dystrophic skeletal [39] and cardiac [17] muscle, corresponding increases in OXPHOS do not appear to occur in dystrophic muscle. In our study, manipulating the [Ca^2+^]_em_ across a 50–200 nM range (Fig. 3) had no effect on MAPR in either control or *mdx* mitochondria. With the exception of relative S+R-stimulated MAPR in TA, in which control and *mdx* MAPR were remarkably comparable, there was a general trend for reduced modulation of MAPR in *mdx* compared to control mitochondria (*p* values ranging from 0.069 to 0.141), however this what not statistically significant. Interestingly, in contrast to the lack of response a 50–200 nM [Ca^2+^] range effected, the addition of 400 nM [Ca^2+^_em_] induced PPKM-stimulated MAPR depression in both control and *mdx* mitochondria from diaphragm and TA (Figs. 3A and 3C), and S+R-stimulated MAPR depression in both control and *mdx* mitochondria from diaphragm (Fig. 3B). While this demonstrates that *mdx* mitochondria are capable of responding to [Ca^2+^]_em_, and, indeed, that they respond in the same way to this “upper resting” load as per normal control mitochondria, general MAPR depression at this concentration is an anomalous finding. Several groups have demonstrated that 400 nM Ca^2+^ is sufficient to induce activation of key regulatory enzymes of OXPHOS including PDH and FAD-glycerol-3-phosphate dehydrogenase (reviewed in [38]), thus a stimulatory effect on MAPR would be expected. Nevertheless, we have shown the same inhibitory effect induced by 400 nM Ca^2+^ in control and *mdx* diaphragm mitochondria for PPKM- and S+R-stimulated MAPR, suggesting that this effect is not substrate specific and therefore, probably not a result of enzymatic PDH inhibition.

Remarkably, we were unable to show 400 nM [Ca^2+^]_em_-induced MAPR inhibition in either control or *mdx* mitochondria from TA, when stimulated with S+R. This would further suggest that diaphragm and TA respond very differently to extra-mitochondrial Ca^2+^ load, and that the target for MAPR inhibition by 400 nM Ca^2+^ may lie somewhere between Complex I and II, since in TA mitochondria MAPR inhibition was observed following PPKM- but not S+R-stimulation. It is interesting to speculate that the inhibitory effect of 400 nM [Ca^2+^]_em_ on S+R-stimulated MAPR in *mdx* diaphragm, but not TA mitochondria may contribute to the more human DMD-like progressive wasting evident in this muscle, especially in the instance that Complex I function is impaired in DMD mitochondria which would increase reliance on FADH_2_-donated H^+^ and electrons through Complex II. A notable difference between mitochondria from the predominantly type I fibre-composed diaphragm and type-II fibre-composed TA is the absence of the mitochondrial calcium binding protein, calmitine. This protein is expressed only in type I fibres [40], although is notably quantitatively reduced in *mdx* mitochondria [41]. It is not known what, if any, role calmitine plays in directly regulating OXPHOS.

In this study, CS analysis of *mdx* muscle was performed to determine the quality of our isolated mitochondria preparations and subsequently normalise MAPR for comparative mitochondrial damage/loss caused by the extraction process and available functionality. While CS_total_ activity was unchanged in dystrophic mitochondria compared to controls in both TA and diaphragm, thus indicating comparable CS expression and mitochondrial content, it was interesting to observe highly significant reductions in the absolute (CS_after_) and functional CS activity (CS_before_) of extracted MS. Taken together with a significant reduction in the CS_after_/CS_total_ ratio and % mitochondrial yield of *mdx* muscle compared to controls, our data suggests that less *mdx* mitochondria are being successfully brought through the extraction process. This finding implicates that dystrophic mitochondria are more susceptible to damage (mechanical or biochemical) during the extraction process, than control mitochondria. Increased fragility of both the inner and outer mitochondrial membranes has been reported previously in mitochondrial fractions derived from gastrocnemius biopsies of DMD patients from as early as one year of age [42] – this is an age at which all muscles show relative stability and normality of function. We have confirmed the same fragility in mitochondria from 12 week old *mdx* mice (Table 1) – this is an age at which the severe cyclical degenerative episodes evident in hind limb muscles earlier in life have attenuated, and the diaphragm has yet to progress to its severe wasting phenotype. Mitochondrial membrane fragility following biochemical isolation is likely associated with the swollen morphology observed in *mdx* mitochondria comparative to controls (Fig. 4) which has also been observed by others [43], and, was apparent both prior to and following the addition of increasing [Ca^2+^] to the extramitochondrial environment. [Ca^2+^] load, however, did not induce any faster swelling of *mdx* mitochondria. This finding is in accordance with Reutenauer et al. [44] who demonstrated comparable responses to Ca^2+^ challenge between biochemically isolated control and *mdx* mitochondria, with the calcium retention capacity and Ca^2+^ overload concentration required to induce permeability transition following repeat 5 µM Ca^2+^ bursts, equivalent [39]. In contrast, Godin et al. [33] has shown a greater susceptibility (decreased time) to permeability transition pore opening and a reduced Ca^2+^_ retention capacity in isolated fibres from the mdx mouse, highlighting differences in the behaviour between *in* *vivo* and *in* *vitro* mitochondrial morphology. Indeed, it is interesting that swelling was persistent despite mitochondria bathing in a low Ca^2+^_em_ environment throughout the isolation procedure, storage and the Ca^2+^-free swelling assay in our study, which suggests that *mdx* mitochondria have a swollen morphological phenotype that is less responsive or unable to respond to subsequent reductions in the concentration of the Ca^2+^_em_ environment. Alternatively, this finding may reflect an artefact of our biochemical isolation technique as subsarcolemmal mitochondrial size is demonstrably comparable between *mdx* and control muscles *in* *vivo*, as detected by transmission electron microscopy [20]. As the subsarcolemmal mitochondrial pool accounts for only ∼10% of total skeletal muscle mitochondria and the comparative morphology of *mdx* intrafibrillar mitochondria (which account for ∼90% of total mitochondria) is yet to be assessed [20], further characterisation of mitochondrial swelling is clearly required.

In summary, we have demonstrated reduced MAPR in mitochondria extracted from diaphragm and TA from the *mdx* mouse model of DMD. As this reduction can be partially ameliorated by bypassing Complex I and directly stimulating Complex II, it suggests that Kreb’s-fuelled NADH-dependent Complex I function is deficient. We have also demonstrated a tendency for MAPR to be modulated differently by the addition of Ca^2+^ to the extra-mitochondrial environment in *mdx* compared to control mitochondria, though in a non-concentration-dependent manner. Further, we have shown depression of PPKM-stimulated MAPR in the presence of 400 nM [Ca^2+^]_em_ in both diaphragm and TA, and S+R-stimulated MAPR in diaphragm in both control and *mdx* mitochondria. We cannot conclude if these findings are reflective of a pre-existing state either inherent to the DMD phenotype or induced by an *in* *vivo* pathophysiological environment that produces persistent morphological maladaptation. As such, further investigation is required to characterise the precise bioenergetical profile of *mdx* mitochondria and the fragility of mitochondrial membranes. Extension of our findings to determine whether Complex I deficiency worsens with age and/or repeat exposure to cyclical damage bouts, is present in other forms of muscular dystrophy characterised by intracellular Ca^2+^ dysregulation, and/or underscores the cardiac muscle pathology evident in DMD, would be of value to the current body of literature.

## Supporting Information

S1 Table **Background ATP production (mmol.min^−1^.intact mitochondrial yield^−1^) of control (c57BL/10) and dystrophic mdx TA and diaphragm.** *p<0.05 *mdx* different from control strain. There was no effect of muscle type (p = 0.323) or extramitochondrial [Ca^2+^] (p = 0.852).
(DOCX)
