# Supplementary material for: Defects in Mitochondrial ATP Synthesis in Dystrophin-Deficient Mdx Skeletal Muscles May Be Caused by Complex I Insufficiency
Source: PLoS One. 2014 Dec 26;9(12):e115763. doi: 10.1371/journal.pone.0115763 (PMC4277356; doi:10.1371/journal.pone.0115763)
Supplement: S1 Table — Background ATP production (mmol.min−1.intact mitochondrial yield−1) of control (c57BL/10) and dystrophic mdx TA and diaphragm. *p<0.05 mdx different from control strain. There was no effect of muscle type (p = 0.323) or extramitochondrial [Ca2+] (p = 0.852). (DOCX) [file pone.0115763.s001.docx]

|  | **TA** | | **DIA** | |
| --- | --- | --- | --- | --- |
|  | **CON** | **MDX** | **CON** | **MDX** |
| 0nM [Ca^2+^] | 0.519 ± 0.725 | 0.125 ± 0.045 ***** | 0.491 ± 0.400 | 0.384 ± 0.052 ***** |
| 50nM [Ca^2+^] | 0.861 ± 0.725 | 0.147 ± 0.061 ***** | 0.412 ± 0.431 | 0.267 ± 0.046 ***** |
| 100nM [Ca^2+^] | 1.733 ± 1.325 | 0.212 ± 0.056 ***** | 0.298 ± 0.387 | 0.243 ± 0.056 ***** |
| 200nM [Ca^2+^] | 1.183 ± 0.790 | 0.186 ± 0.056 ***** | 0.393 ± 0.472 | 0.322 ± 0.075 ***** |
| 400nM [Ca^2+^] | 1.594 ± 1.051 | 0.209 ± 0.059 ***** | 1.077 ± 0.399 | 0.317 ± 0.268 ***** |

TABLE S1. Background ATP production (mmol.min^-1^_._.intact mitochondrial yield^-1^) of control (c57BL/10) and dystrophic mdx TA and diaphragm. *p<0.05 *mdx* different from control strain. There was no effect of muscle type (p=0.323) or extramitochondrial [Ca^2+^] (p=0.852).
